# MafB deficiency accelerates the development of obesity in mice

**DOI:** 10.1002/2211-5463.12058

**Published:** 2016-04-21

**Authors:** Mai Thi Nhu Tran, Michito Hamada, Megumi Nakamura, Hyojung Jeon, Risa Kamei, Yuki Tsunakawa, Kaushalya Kulathunga, Yuan‐Yu Lin, Kumiko Fujisawa, Takashi Kudo, Satoru Takahashi

**Affiliations:** ^1^ Department of Anatomy and EmbryologyFaculty of MedicineUniversity of TsukubaIbarakiJapan; ^2^ Laboratory Animal Resource CenterFaculty of MedicineUniversity of TsukubaIbarakiJapan; ^3^ International Institute for Integrative Sleep Medicine (WPI‐IIIS)University of TsukubaIbarakiJapan; ^4^ Ph.D. Program in Human BiologySchool of Integrative and Global MajorsUniversity of TsukubaIbarakiJapan; ^5^ Laboratory of Molecular BiologyDepartment of Animal Science and TechnologyNational Taiwan UniversityTaipeiTaiwan

**Keywords:** adipose tissue macrophages, apoptosis inhibitor of macrophage, MafB, obesity

## Abstract

MafB, a transcription factor expressed selectively in macrophages, has important roles in some macrophage‐related diseases, especially in atherosclerosis. In this study, we investigated the mechanism by which hematopoietic‐specific MafB deficiency induces the development of obesity. Wild‐type and hematopoietic cell‐specific *Mafb*‐deficient mice were fed a high‐fat diet for 10 weeks. The *Mafb*‐deficient mice exhibited higher body weights and faster rates of body weight increase than control mice. The *Mafb*‐deficient mice also had a higher percentage of body fat than the wild‐type mice, due to increased adipocyte size and serum cholesterol levels. Reverse transcription‐PCR analysis showed a reduction in apoptosis inhibitor of macrophage (AIM) in *Mafb*‐deficient adipose tissue. AIM is known as an inhibitor of lipogenesis in adipocytes and is expressed in adipose tissue macrophages. Collectively, our data suggest that *Mafb* deficiency in hematopoietic cells accelerates the development of obesity.

**Abbreviations**
AIM: apoptosis inhibitor of macrophage
ATM: adipose tissue macrophage
CT: computed tomography
GFP: green fluorescent protein
HE: hematoxylin and eosin
HFD: high‐fat diet
KO: knock‐out
LDL: low‐density lipoprotein
LXR: liver X receptor
Maf: musculoaponeurotic fibrosarcoma
MARE: Maf recognition element
WT: wild‐type

MafB, also known as v‐maf musculoaponeurotic fibrosarcoma oncogene homolog B, belongs to the large Maf transcription factor family, the members of which contain basic leucine zipper (bZIP) domains that bind to DNA. These bZIP domains are located within the Maf recognition element (MARE) and regulate the transcription of target genes by binding to acidic domains within the genes 1. MafB plays various roles in a variety of differentiation processes, including the differentiation of pancreatic α and β cells, podocytes in the renal glomerulus, and rhombomeres (r5) in the embryonic hindbrain. MafB is also central to embryonic thymus development, parathyroid gland development, the creation of hair cuticles, and urethral masculinization 2, 3, 4, 5, 6, 7. In the hematopoietic system, MafB is important for myeloid lineage commitment of hematopoietic stem cells and for macrophage differentiation 5, 8. Recently, we reported that MafB expression is induced by liver X receptor (LXR), which is activated by oxidized low‐density lipoprotein (LDL) and regulates apoptosis inhibitor of macrophage (AIM, also called Api6/Spα/CD5L). Consistently, MafB deficiency has been shown to ameliorate atherosclerotic lesions due to increased apoptosis of foam cells 9.

Apoptosis inhibitor of macrophage, a member of the scavenger receptor cysteine‐rich superfamily, is a soluble protein mainly expressed in macrophages. AIM has multiple functions, including inhibiting the induction of apoptosis, inducing the coagulation of certain types of bacteria, and preventing the development of autoimmunity by binding to IgM 10, 11, 12, 13, 14. AIM expression in adipose tissue macrophages (ATMs) plays a role in adipocyte lipolysis. AIM is incorporated into adipocytes via CD36 scavenger receptor‐mediated endocytosis. Following this, AIM binds to fatty acid synthase (FASN), thereby inhibiting its activity 15, 16. *AIM‐*deficient mice develop obesity and increase adipose tissue mass 16. Although MafB regulates AIM in foam cells within atherosclerotic lesions, it is unknown whether MafB also regulates AIM in ATMs.

In this study, we investigated the relationship between MafB and obesity using the following two approaches: (1) we reconstituted hematopoietic systems in mice via transplantation of *Mafb* ^*−/−*^ fetal liver cells and fed these mice a high‐fat diet (HFD), and (2) we fed a HFD to hematopoietic cell‐specific *Mafb*‐deficient mice (*Mafb* ^*f/f*^::Tie‐2‐Cre). *Mafb*‐deficient mice exhibited increases in body weight and adipose tissue weight. Quantitative RT‐PCR results showed that *AIM* expression decreased in *Mafb*‐deficient adipose tissue. Our results indicate that MafB inhibits increases in fat mass under HFD conditions.

## Materials and methods

### Mice

*Mafb* ^*−/−*^mice were generated in a 129/Sv background and backcrossed to the C57BL/6J strain for over seven generations 5. The primer sequences used for genotyping were described in previous studies 5. For hematopoietic system reconstitution, fetal liver cells were isolated from either E14.5 wild‐type or *Mafb* ^*−/−*^ (C57BL/6J‐Ly5.1) embryos. Following this, lethally irradiated (7 Gy) 6‐week‐old wild‐type (C57BL/6J‐Ly5.2) mice were injected with 5 × 10^6^ fetal liver cells per mouse via the tail vein. Donor cell chimerism was determined based on the Ly5.1^+^/(Ly5.1^+^ + Ly5.2^+^) cell ratio. Mice with > 95% chimerism were used in further experiments. To create *Mafb* conditional knock‐out (KO) mice, the *Mafb* gene was flanked with a loxP element with a neomycin‐resistant gene using homologous recombination in ES cells (*Mafb* ^*f/f*^). Following this, the mice were crossed with Tie2‐Cre knock‐in mice (*Mafb* ^*f/f*^::Tie2‐Cre mice). To induce obesity, the mice were fed a HFD for 10 weeks. Each week, the body weight of mice and their food consumption were measured. The mice were maintained under specific pathogen‐free conditions in a laboratory animal resource center at the University of Tsukuba. All experiments were performed according to the Guide for the Care and Use of Laboratory Animals of University of Tsukuba.

### Computed tomography scanning

To evaluate the body fat percentage in mice, we performed computed tomography (CT) scanning. Mice were anesthetized via intraperitoneal injection of a mixture of medetomidine (0.3 mg·kg^−1^; Zenoaq, Fukushima, Japan), midazolam (4 mg·kg^−1^; Astellas, Tokyo, Japan), and butorphanol (5 mg·kg^−1^; Meiji Seika Pharma, Tokyo, Japan). Following this, their body fat percentage was measured using an Aloka LCT‐100A CT Scanner.

### Serum cholesterol measurement

To collect serum, whole blood was collected and stored at room temperature for 30 min. Following this, the blood was centrifuged for 30 min at 2000 ***g*** at 4 °C, and the separated serum was transferred into new tubes. Cholesterol levels were measured using an Automated Clinical Chemistry Analyzer (DRI‐CHEM 7000V; Fujifilm, Tokyo, Japan).

### Histological analysis

In this study, we utilized male mice for the hematopoietic system reconstitution and female mice for conditional KO. Epidermal fat pads were collected from wild‐type and *Mafb* ^*−/−*^ mice, and inguinal and ovary fat pads were collected from *Mafb* ^*f/f*^ and *Mafb* ^*f/f*^::Tie2‐Cre mice. The collected fat pads were either fixed in 4% paraformaldehyde solution and embedded in optimum cutting temperature compound or fixed in neutral‐buffered formalin and embedded in paraffin. Paraffin sections were stained with hematoxylin and eosin (HE), and adipocyte size was measured using imagej analysis software (NIH, Bethesda, MD, USA). The frozen sections were incubated with rabbit anti‐GFP (MBL, Woburn, MA, USA), rabbit anti‐(mouse MafB) (Bethyl, Montgomery, TX, USA) and rat anti‐(mouse Mac‐2) (Cedarlane, Burlington, NC, USA). Alexa Fluor 488‐conjugated goat anti‐(rabbit IgG) (Molecular Probes, Eugene, OR, USA) and Cy3‐conjugated donkey anti‐(rat IgG) (Jackson ImmunoResearch, West Grove, PA, USA) were used as secondary antibodies. Hoechst 33342 (Thermofisher, Rockford, IL, USA) was used to stain nuclei.

### Quantitative RT‐PCR analysis

Adipose tissue was collected and incubated with 0.5% collagenase A (Roche, Mannheim, Germany) for 1 h at 37 °C. The digested adipose tissue was filtered through a 70‐μm strainer and centrifuged at 300 ***g*** for 4 min at 4 °C. The cell pellet was collected and washed with phosphate‐buffered saline. Total RNA was collected using an Isogen kit (Nippon gene, 311‐02501). cDNA was synthesized using a QuantiTect Reverse Transcription Kit (Qiagen Valencia, CA, USA). The mRNA levels of *Mafb*,* AIM, and Mac‐1* were measured using SYBR green PCR master mix (Takara Bio, Otsu, Japan). The mRNA levels of *Mafb* and *AIM* were normalized to the *Mac‐1* mRNA level. The following primer sequences were used: *Mafb* forward, 5′‐TGAATTTGCTGGCACTGCTG‐3′; *Mafb* reverse, 5′‐AAGCACCATGCGGTTCATACA‐3′;*AIM* forward, 5′‐GTACCACGACTGTACCCACAAGGA‐3′;*AIM* reverse, 5′‐GAATGAGGGCCCACTGAACAA‐3′; *Mac‐1*forward, 5′‐ATGGACGCTGATGGCAATACC‐3′; *Mac‐1* reverse, 5′‐ TCCCCATTCACGTCTCCCA‐3′;

### Statistical analysis

All data are shown as the mean ± SEM. *P*‐values were calculated using two‐tailed Student's *t*‐tests.

## Results

### MafB affects body weight in mice

**Figure 1:**
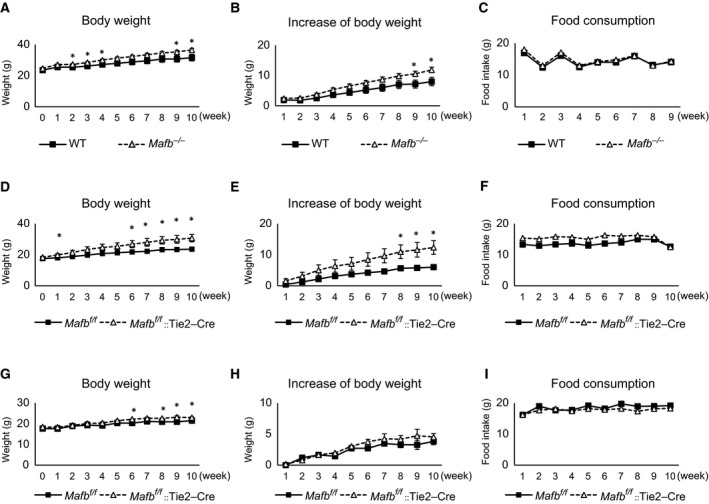
MafB deficiency accelerates the development of obesity. (A–C) Wild‐type (WT) and *Mafb* ^*−/−*^ mice fed with a high‐fat diet (HFD). (D–F) *Mafb* ^*f/f*^ and *Mafb* ^*f/f*^::Tie2‐Cre mice fed a HFD. (G–H) *Mafb* ^*f/f*^ and *Mafb* ^*f/f*^::Tie2‐Cre mice fed a normal diet. (A, D, and G) Body weight. (B, E, and H) Rate of body weight increase. (C, F, and I) Food consumption. All data represent the means ± SEM. *P < 0.05 (Student's *t*‐test). WT,* n* = 6; *Mafb* ^*−/−*^, *n* = 11; *Mafb* ^*f/f*^, *n* = 8; *Mafb* ^*f/f*^::Tie2‐Cre, *n* = 8.

Because *Mafb* deficiency strongly reduced the expression of AIM both in foam cell within atherogenic lesion and *in vitro* macrophages 9. A Recent report identified that AIM‐deficient mice developed obesity, because AIM inhibited adipose tissue mass in high‐fat diet (HFD) condition 16. As this paper showed that the cells that express AIM were adipose tissue macrophages, we hypothesized that MafB is also expressed in adipose tissue macrophages and inhibits adipose tissue mass through regulation of AIM expression. To address this hypothesis, first we examined the effects of MafB on mouse body weight in HFD condition. Because *Mafb*‐deficient mice die after birth 5, we transplanted fetal liver cells from wild‐type and *Mafb* ^*−/−*^ E14.5 embryos into X‐ray‐irradiated recipient mice to generate wild‐type and *Mafb* ^*−/−*^ hematopoietic system‐reconstituted mice. Following this, the mice were fed a HFD for 10 weeks, and their body weights were measured. After 9 weeks, the *Mafb* ^*−/−*^ mice had significantly higher body weights compared to the wild‐type mice (Fig. 1A). The body weights of the *Mafb* ^*−/−*^ mice also increased at a higher rate than those of the wild‐type mice (Fig. 1B). In addition, we also generated *Mafb* conditional KO mice, *Mafb* ^*f/f*^::Tie2‐Cre mice, that *Mafb* expression was specifically lacked in hematopoietic stem cells and macrophages 17, 18, 19. After feeding these mice a HFD for 6 weeks, their body weights and rates of body weight increase were both higher than those of the *Mafb* ^*f/f*^ mice (Fig. 1D,E). Food consumption did not differ between the groups (Fig. 1C,F). Moreover, when fed a normal diet, we found that the body weights of the *Mafb* ^*f/f*^::Tie2‐Cre mice only slightly increased while the difference in body weight gain between the *Mafb* ^*f/f*^::Tie2‐Cre mice and wild‐type mice was not detected, and the food consumption of both groups was similar (Fig. 1G–I). Taken together, these data suggest that MafB deficiency accelerates weight gain in mice.

### MafB affects fat storage in mice

**Figure 2:**
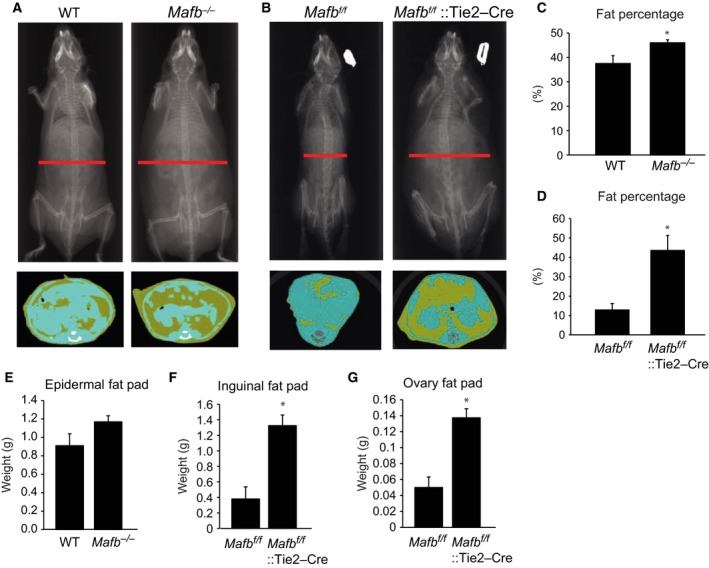
MafB deficiency increases fat storage in mice fed a high‐fat diet (HFD). (A) Computed tomography (CT) images of wild‐type (WT) and *Mafb* ^*−/−*^ mice. (B) CT images of *Mafb* ^*f/f*^ and *Mafb* ^*f/f*^::Tie2‐Cre mice. (A, B) Upper panel, Radiograph of the axial sleleton of mice; Lower panel, Representative CT transxial images (yellow parts: fat); The red lines, position of the representative CT transxial images. (C) Body fat percentages in WT and *Mafb* ^*−/−*^ mice. (D) Body fat percentages in *Mafb* ^*f/f*^ and *Mafb* ^*f/f*^::Tie2‐Cre mice. (E) Epididymal fat pad weights in WT and *Mafb* ^*−/−*^ mice. (F) Inguinal fat pad weights in *Mafb* ^*f/f*^ and *Mafb* ^*f/f*^::Tie2‐Cre mice. (G) Ovary fat pad weights in *Mafb* ^*f/f*^ and *Mafb* ^*f/f*^::Tie2‐Cre mice. All data are shown as the means ± SEM. *P < 0.05 (Student's *t*‐test). WT,* n* = 6; *Mafb* ^*−/−*^, *n* = 11; *Mafb* ^*f/f*^, *n* = 4; *Mafb* ^*f/f*^::Tie2‐Cre, *n* = 4.

**Figure 3:**
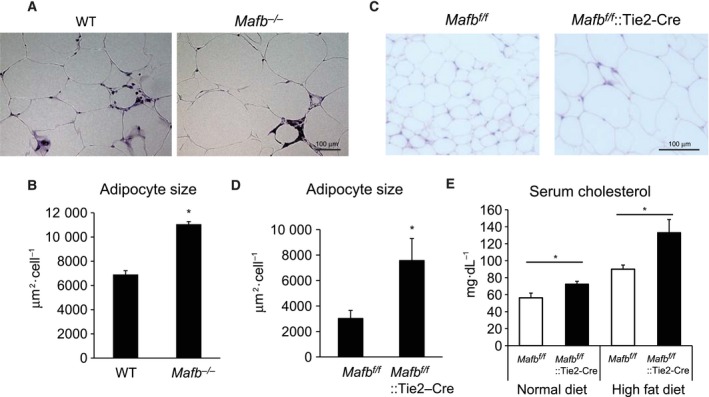
MafB deficiency increases adipocyte sizes and serum cholesterol levels in mice fed a high‐fat diet (HFD). (A) Hematoxylin & eosin (HE)‐stained adipose tissues from wild‐type (WT) and *Mafb* ^*−/−*^ mice. (B) Adipocyte sizes in WT and *Mafb* ^*−/−*^ mice. (C) HE‐stained adipose tissues from *Mafb* ^*f/f*^ and *Mafb* ^*f/f*^::Tie2‐Cre mice. (D) Adipocyte sizes in *Mafb* ^*f/f*^ and *Mafb* ^*f/f*^::Tie2‐Cre mice. (E) Serum cholesterol levels in *Mafb* ^*f/f*^ and *Mafb* ^*f/f*^::Tie2‐Cre mice fed a normal diet and a HFD. All data are shown as the means ± SEM. *P < 0.05 (Student's *t*‐test). WT,* n* = 6; *Mafb* ^*−/−*^, *n* = 11; *Mafb* ^*f/f*^, *n* = 4; *Mafb* ^*f/f*^::Tie2‐Cre, *n* = 4.

Next, we used CT scanning to measure fat storage (Fig. 2A,B, upper panels). The CT transaxial images showed that the *Mafb* ^*−/−*^ and *Mafb* ^*f/f*^::Tie2‐Cre mice both had higher body fat accumulations (yellow parts) than the control mice (Fig. 2A,B, lower panels). Furthermore, the whole body fat percentages of the *Mafb* ^*−/−*^ and *Mafb* ^*f/f*^::Tie2‐Cre mice were also higher than those of control mice (Fig. 2C,D). In addition, we collected epididymal fat pads from the wild‐type and *Mafb* ^*−/−*^ mice and inguinal and ovary fat pads from the *Mafb* ^*f/f*^ and *Mafb* ^*f/f*^::Tie2‐Cre mice and measured the fat pad weights. The data showed that the epididymal fad pads from the *Mafb* ^*−/−*^ mice tended to have higher weights than those of the wild‐type mice (Fig. 2E). The *Mafb* ^*f/f*^::Tie2‐Cre mice had significantly heavier inguinal and ovary fat pads than the *Mafb* ^*f/f*^ mice (Fig. 2F,G). We also performed HE staining and measured adipocyte sizes. The *Mafb*‐deficient mice had larger adipocytes than the wild‐type mice (Fig. 3A,B). The *Mafb* ^*f/f*^::Tie2‐Cre mice had larger adipocytes than the *Mafb* ^*f/f*^ mice (Fig. 3C,D). Moreover, the *Mafb* ^*f/f*^::Tie2‐Cre mice had higher serum cholesterol levels than the *Mafb* ^*f/f*^ mice in both the normal diet and HFD groups (Fig. 3E). These data indicate that MafB deficiency increases the fat storage and serum cholesterol levels in mice. Because both of the evaluated *Mafb*‐deficient lines (*Mafb* ^*f/f*^::Tie2‐Cre and mice reconstituted with *Mafb* ^*−/−*^ fetal liver cells) showed a lack of MafB in hematopoietic cells only, we hypothesized that the presence of MafB in hematopoietic cells, such as macrophages, is responsible for adipose tissue mass.

### MafB deficiency reduces *AIM* expression in adipose tissue macrophages

**Figure 4:**
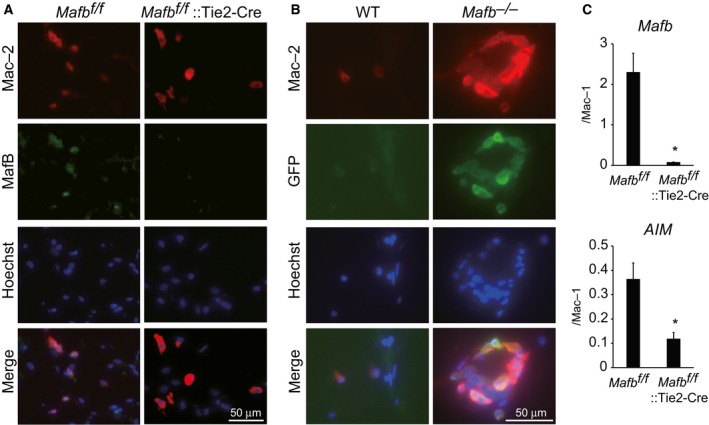
MafB deficiency reduces the expression of *AIM* in adipose tissue macrophages. (A) *Mafb* ^*f/f*^ and *Mafb* ^*f/f*^::Tie2‐Cre adipose tissue sections stained with antibodies against MafB and Mac‐2. (B) Wild‐type (WT) and *Mafb* ^*−/−*^ adipose tissue sections stained with antibodies against GFP and Mac‐2. (C) *Mafb* and *AIM* mRNA expression measured by qRT‐PCR. All data are shown as the means ± SEM. *P < 0.05 (Student's *t*‐test). WT,* n* = 3; *Mafb* ^*−/−*^, *n* = 3; *Mafb* ^*f/f*^, *n* = 3; *Mafb* ^*f/f*^::Tie2‐Cre, *n* = 4.

Previous reports have shown that ATMs are associated with development of obesity 20, 21, 22. Because MafB plays an important role in the development and functioning of macrophages 5, 9, we characterized *Mafb* expression in ATMs. We collected fat pads from mice of each genotype and made frozen sections. Then adipose sections were stained with anti‐MafB antibody and anti‐Mac‐2 antibody, which has been previously reported to be expressed in ATMs 23, 24. The data showed that MafB expression was detected in Mac‐2 positive *Mafb* ^*f/f*^ macrophages which indicated MafB is expressed in adipose tissue macrophages (Fig. 4A). In contrast, signals of MafB are not detected in *Mafb* ^*f/f*^::Tie2‐Cre mice. Moreover, we examined immunostaining using anti‐GFP antibody and anti‐Mac‐2 antibody. *GFP* gene was inserted into *Mafb* locus in *Mafb*‐deficient mice 5. The GFP expression derived by *Mafb* gene promoter was detected in Mac‐2‐positive macrophages (Fig. 4B), implying a functional role of MafB in ATMs.

It has been previously reported that *AIM*‐deficient mice exhibit increased adipocyte size and body fat weight when fed a HFD 16, 25. We have previously demonstrated that MafB directly regulates *AIM* expression in foam cells in atherosclerosis 9. Therefore, we investigated *AIM* expression in *Mafb* ^*−/−*^ macrophages. We found that both *Mafb* and *AIM* expression were significantly decreased in *Mafb* ^*f/f*^::Tie2‐Cre mice relative to *Mafb* ^*f/f*^ mice (Fig. 4C). These results indicate that reducing the expression of AIM in *Mafb*‐deficient ATMs might affect adipose tissue mass and induce obesity.

## Discussion

In this study, we first demonstrated that MafB deficiency in the hematopoietic system accelerates weight gain, enhances the body fat storage, and increases the adipocyte size. In adipose tissue, the reduction in *AIM* expression was observed in *Mafb*‐deficient mice (Fig. 4C). As the *AIM* promoter region contains a MARE and MafB binds to this site in macrophage within atherosclerosis lesion 9, it is possible that MafB directly regulates *AIM* expression in ATMs. Recent studies have shown that AIM is also important for inhibiting obesity‐related autoimmunity and steatosis‐associated hepatocellular carcinoma tumor development 26, 27. Further analysis is required to determine whether MafB regulates *AIM* expression in these cases.

Our previous study showed human MAFB and AIM were coexpressed in atherosclerosis lesion of patient 9. In addition, a recent study showed that human MAFB is expressed in ATMs and MAFB expression correlates negatively with lipogenesis and lipolysis in human adipocytes 28. These evidence suggest that MAFB may regulate AIM expression in human ATMs because AIM also inhibits fatty acid synthase activity of adipocyte 16.

Both in human and mouse, MafB is regulated by nuclear receptor transcription family such as liver x receptor (LXR), retinoic acid receptor (RAR), or retinoid x receptor (RXR) in monocytes/macrophages 9, 29. Especially, LXR regulates AIM through MafB regulation in macrophage within atherosclerosis lesion 9. Further analysis is required to find which pathway induces MafB expression in ATMs.

Overall, we showed that *Mafb*‐deficient mice revealed an obesity phenotype. These mice might be useful as models in the investigation of new treatments for obesity.

## Author contributions

MTNT, MH, RK, HJ, and MN performed mouse experiments. MTNT and MN performed histological experiments. MTNT and RK performed qRT‐PCR analysis. YT performed CT scanning. KK and YL measured adipocyte size. MTNT and KF generated the *Mafb* conditional KO mice. MH, TK, and ST contributed to hypothesis development, experimental design, and data interpretation.
